# Successful Use of Eltrombopag in a Young Child With Chronic Immune Thrombocytopenia

**DOI:** 10.7759/cureus.12723

**Published:** 2021-01-15

**Authors:** Bernd Gruhn, Achim Ehrnsperger, Claudia Willy, Birgit Fröhlich

**Affiliations:** 1 Department of Pediatrics, Jena University Hospital, Jena, DEU; 2 Translational Clinical Oncology & Mature Products, Novartis Pharma GmbH, Nürnberg, DEU; 3 Department of Pediatrics, Münster University Hospital, Münster, DEU

**Keywords:** chronic immune thrombocytopenia, child, thrombopoietin receptor agonist, eltrombopag

## Abstract

The disease course in a young girl with chronic immune thrombocytopenia (ITP) at the initial age of 19 months, treated with eltrombopag, was evaluated retrospectively and is presented as a case record and discussed against the background of the available literature. A stable response and reduction in clinical symptoms, over several years and without frequent dose changes, was achieved. Bleeding symptoms were mild to moderate and occurred particularly frequently in combination with low platelet counts. Raising the platelet count, in turn, was accompanied by a decreased bleeding tendency. Eltrombopag was tolerated well. No new safety signals were observed during the treatment. Based on a follow-up of more than 2.5 years, our case confirms that a child with chronic ITP can benefit from treatment with eltrombopag in the regular care setting. We assume that early treatment with a thrombopoietin receptor agonist could save many children from repeated and lengthy hospitalizations with intravenous immunoglobulins and prolonged administration of corticosteroids.

## Introduction

Immune thrombocytopenia in children

Immune thrombocytopenia (ITP) is an acquired disease characterized by specific antibodies against platelets and megakaryocytes, leading to increased degradation of thrombocytes, insufficient thrombocytopoiesis, and a relative thrombopoietin deficiency. With platelet counts lower than 10-30 × 10^9^/L, an increased bleeding risk can be expected. However, there is a high interindividual variance [1]. The incidence rate of ITP in children is estimated at 0.2-0.7/10,000/year, which appears to be similar in adults. However, due to the higher proportion of cases with short disease duration, prevalence is lower than that in adults: in children, it is approximately 0.4-0.5/10,000/year and in adults 0.9-2.6/10,000/year [1-3]. In children, ITP often occurs subsequent to a viral infection and rarely after administering a live attenuated vaccine. Spontaneous remission rates are high [4-5]. In observational studies, approximately two-thirds of all patients reached spontaneous remission within six months [4,6-8]. In 20-30% of all such cases, the disease persists longer than 12 months, fulfilling the diagnostic criterion for chronic ITP [2,4,9,10]. Severe bleeding events are rare in children with ITP. Even with platelet counts of <20 × 10^9^/L, most of the cases present with only mild-to-moderate bleeding of skin and mucosa (World Health Organization grade I-II), without requiring medical intervention. A systematic review found that the weighted proportion for intracranial hemorrhage was 0.4% for children (95% confidence interval, 0.2-0.7%), most of whom had chronic ITP [11].

Therapeutic strategies

In addition to a wait-and-watch strategy in patients with mild-to-moderate bleeding symptoms, corticosteroids and intravenous immunoglobulins (IVIg) are used as first-line medication in children with chronic ITP [9,12]. For second-line therapy, thrombopoietin receptor agonists (TPO-RA) are recommended. Even though splenectomy is an effective treatment of ITP, in children it is considered as a last resort because of the associated risk for infections with encapsulated bacteria and the lifelong risk of overwhelming postsplenectomy infection syndrome. The younger the splenectomized child, the higher is the risk of infection. According to the German guidelines related to newly diagnosed ITP in children and adolescents, the only indication for splenectomy is emergency treatment of a non-controllable hemorrhage [12]. If therapy fails again or if recurrences occur, further treatment options include rituximab as a monoclonal anti-CD20 antibody, immunosuppressive drugs, or a combination of selected therapies [9]. In ITP patients, platelet transfusions should only be considered in case of severe bleeding.

Treatment with eltrombopag

The efficacy, tolerability, and safety of TPO-RA have been proven in children with chronic ITP by randomized controlled trials [13-15]. The two international trials PETIT [16]‎ and PETIT2 [17] are relevant to the TPO-RA eltrombopag. They included a total of 171 ITP patients aged 1-17 years. A pooled analysis of both trials showed that 62% of the patients treated for six weeks with eltrombopag achieved a response compared to 24% of the placebo group. Response was defined as an increase in platelet count of at least 50 × 10^9^/L. Bleeding risk was significantly lower with eltrombopag than with placebo [18]. Adverse event rates in the PETIT studies showed no significant difference between placebo and eltrombopag groups. The most common adverse events with eltrombopag were headache, upper respiratory infections, and nasopharyngitis. In the randomized phase, an increase in alanine aminotransferase (ALT) was noted in five patients (4.7%) in the eltrombopag group and in none of patients in the placebo group. ALT normalized in all affected patients, either during treatment or after discontinuation of eltrombopag [18]. As eltrombopag has a chelating effect on bivalent cations, it can bind intracellular and extracellular iron. In a monocentric retrospective series of cases including 11 patients with an average age of 12 years (range: 8-16), a significant decrease in serum ferritin and mean corpuscular volume was observed with eltrombopag over a three-month period. Three patients developed iron deficiency anemia [19]. In pivotal studies, no iron deficiencies have emerged with eltrombopag. However, the corresponding laboratory parameters were not regularly tested in these studies. In experimental studies, eltrombopag crossed the blood-brain barrier and inhibited iron-dependent maturation and differentiation of neurons and dendrites in the early stages of development [20]. According to German guidelines, indication for the use of the drug should be applied carefully, including a comprehensive developmental neurological (post-) examination [12]. Here, we report on the course and outcome of chronic ITP in a young girl treated with eltrombopag. Our case is from the Department of Pediatrics, Jena University Hospital. The therapy with eltrombopag was initiated in this hospital, while the pretreatment was partially performed at other centers.

## Case presentation

Initial clinical presentation

**Figure 1:**
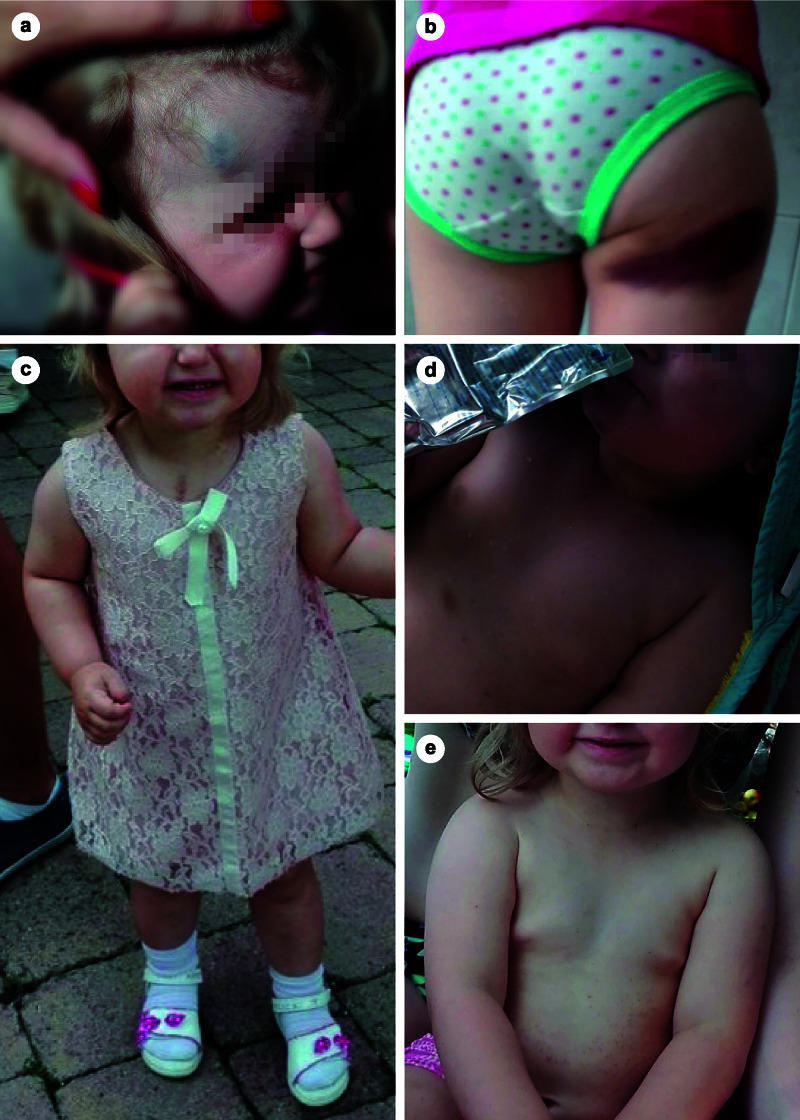
Hematomas occurred repeatedly throughout the body surface, for example, on the temples (a) and back of the thigh (b). In addition, multiple hematomas on the face, trunk, and extremities (c, d) and petechiae on the trunk (e) were observed.

In February 2015, a 19-month-old girl was admitted to the Department of Pediatrics of a regional hospital. For several weeks, the mother had observed “red spots” on the child’s body. In addition, an increasing number of hematomas were present throughout the body surface. Approximately one week before the first symptoms appeared, the girl had a respiratory infection. The initial examination showed numerous petechiae on the neck, trunk, and extremities, as well as multiple hematomas on the face, trunk, and extremities (Figure 1). In the left buccal cavity, a mucosal bleeding of approximately 0.5 cm was visible. The girl showed signs of an infection of the upper airways with serous rhinitis. During the laboratory analysis, significantly reduced platelet counts were noticeable: platelet count in ethylenediaminetetraacetic acid whole blood and citrate blood was 0 × 10^9^/L and 2 × 10^9^/L, respectively.

Pretreatment

Based on an isolated thrombocytopenia with above-mentioned bleeding signs, diagnosis of ITP was made and treatment with corticosteroids was initiated. With dexamethasone, platelet counts in our case could only be temporarily increased to maximum values of 10-13 × 10^9^/L. Two weeks later, the patient received a single dose of immunoglobulin 0.5 g/kg intravenously. Dexamethasone was continued in descending doses. On the first day after treatment with IVIg, the platelet count was 148 × 10^9^/L, which at discharge from the hospital was 183 × 10^9^/L. Three days later, new hematomas were observed on the girl’s forehead, on the left lower leg, and in the area of the central venous catheter puncture site in the right groin. The platelet count was 5 × 10^9^/L. The girl received IVIg again. The platelet count then increased to 15 × 10^9^/L. Thereafter, petechiae and hematomas as well as occasionally mild nosebleeds occurred repeatedly. After four weeks and at a platelet count of 2 × 10^9^/L, the patient received her third IVIg treatment and additional prednisolone due to the lack of an increase in platelets. Finally, the platelet count increased to 29 × 10^9^/L. In November 2015, the girl experienced recurrent abdominal pain and two events of visible blood in the stool, presumably due to a small anal fissure. She also had pronounced petechiae on the trunk, multiple hematomas above the spine, hip, and lower extremities. The platelet count was 3 × 10^9^/L. Because of otitis media, the patient received amoxicillin. Thereafter, the ITP remained untreated.

Investigations

For the treatment of ITP as well as the diagnostic clarification of a newly occurring leukopenia (leukocytes 3.5 × 10^9^/L), one year later the patient was admitted for the first time to the Department of Pediatrics at the Jena University Hospital. Since the middle of the year, petechiae had again appeared on the trunk. The bone marrow smear showed no evidence of malignant or myeloproliferative disease. Further laboratory examination, for example, functional parameters of the liver and kidney, differential blood count and flow cytometry, immunoglobulins, ferritin, tumor markers, and serological tests for several infectious diseases showed normal findings. Neither in clinical examination nor in laboratory testing any evidence for other causes of thrombocytopenia or other differential diagnoses important to consider was noted, such as systemic lupus erythematosus, chonic inflammatory bowel disease, or von Willebrand disease. The platelet count at admission was 9 × 10^9^/L, which increased after IVIg treatment to 103 × 10^9^/L, and within four weeks decreased again to 45 × 10^9^/L. Since mid-December, petechiae reappeared on the trunk. The examination also revealed petechiae on the palate.

Treatment

**Figure 2:**
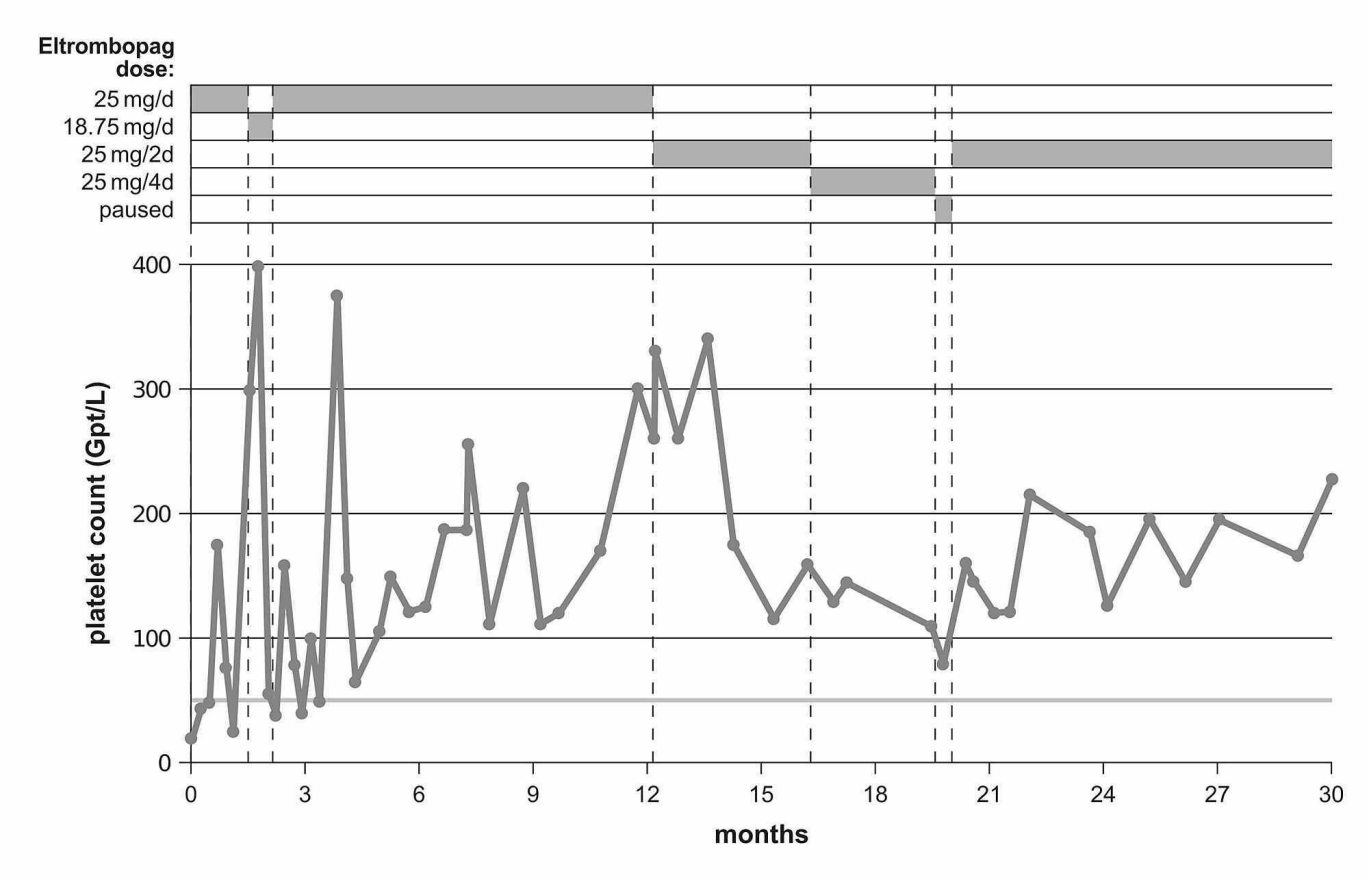
Variation of platelet counts. The gray rectangles above show the respective eltrombopag dosages within the indicated time periods.

At the end of December, 2016, the girl’s parents were informed about the various treatment options. They were told that a therapeutic attempt with eltrombopag, which has been approved for ITP in children, was suitable. Treatment was started with eltrombopag 25 mg/day. The platelet count on the day of treatment initiation was 20 × 10^9^/L. The patient tolerated the treatment with eltrombopag very well. During the first two weeks of treatment, the platelet count increased to 48 × 10^9^/L (for complete course of platelet counts during eltrombopag treatment, see Figure 2), and the petechiae disappeared completely. Within the first three months of treatment with eltrombopag, platelet values were subjected to large fluctuations from minimum values of approximately 25 × 10^9^/L to maximum values of approximately 400 × 10^9^/L. Reduction of the daily dosage of eltrombopag to 18.75 mg reduced the platelet counts below 40 × 10^9^/L. Therefore, the dosage of eltrombopag was increased again to 25 mg/day. During the following nine months, platelet counts varied between 55 and 375 × 10^9^/L. Mild hematomas occurred occasionally, for example, after falls. No more petechiae were observed. With stepwise dose reductions, platelet counts could be maintained between 100 and 300 x 10^9^/L.

Outcome and follow-up

By mid-August, 2018, 19 months after initiation of eltrombopag therapy, the patient was asymptomatic and eltrombopag was discontinued. Nine days later, the platelet count was 79 × 10^9^/L. Treatment with eltrombopag was resumed at a dosage of 25 mg every two days. During the following 12 months, the platelet count was above 100 × 10^9^/L and increased to 242 × 10^9^/L. The patient remained symptom free. The parameters of liver and kidney function as well as iron metabolism were checked periodically during the entire course of treatment and were without any pathological results.

## Discussion

Our case report confirms that the efficacy, tolerability, and safety of treatment of chronic ITP in children, as proven in randomized controlled trials, applies to scenarios of regular care beyond clinical studies, and that under given conditions, patients can benefit significantly from treatment with eltrombopag. The objective of a stable response, with platelet counts above 50 × 10^9^/L and a reduction in clinical symptoms for more than 2.5 years without frequent dose adjustments, was achieved in our case. Overall, platelet counts below 10 × 10^9^/L were associated with a more pronounced bleeding symptomatology, while the symptoms disappeared at stable values of >50 × 10^9^/L on treatment with eltrombopag. No severe bleeding events occurred, even in situations with very low platelet counts. Treatment indication was established in close consultation with the affected girl and her parents. Eltrombopag was very well tolerated. Side effects did not occur. No new signals regarding the safety of eltrombopag were registered. The girl in our case report showed no evidence of developmental neurological impairments during the two-year treatment with eltrombopag. Ferritin was regularly controlled in the child and remained stable during treatment with eltrombopag. We did not find any evidence of iron deficiency anemia in the blood count.

Our case report also confirms the benefits of the oral administration of eltrombopag. The fact that there is no need for injections is particularly child-friendly and in some cases can increase the willingness of children to agree to a necessary treatment plan. In addition, the dosage is better controllable in the short term with a tablet to be taken once daily or a suspension prepared from powder compared to once weekly subcutaneous injections.

In our opinion, as described, the advantages of eltrombopag justify the use of this relatively expensive drug over other treatment options in children with ITP. We assume that early treatment with a TPO-RA can save many children from repeated and lengthy inpatient treatment with IVIg and corticosteroids. Subsequently, this would also pay off in terms of cost-effectiveness. Further studies to evaluate these relationships are needed.

## Conclusions

In summary, the efficacy, tolerability, and safety of eltrombopag in the treatment of chronic ITP has been confirmed in this child over a long period of follow-up. However, the large heterogeneity of the course of treatment, which has been shown in controlled clinical trials as well as in larger cohort studies, underlines the urgency of finding meaningful predictors capable of assessing at an early stage the chances of a response to treatment in the specific case. The patient-friendly way of application as well as the high tolerability of eltrombopag facilitates the decision to try a treatment with this drug even in borderline cases.
